# Supplementary material for: A functional screen uncovers circular RNAs regulating excitatory synaptogenesis in hippocampal neurons
Source: Nat Commun. 2025 Mar 28;16:3040. doi: 10.1038/s41467-025-58070-4 (PMC11953392; doi:10.1038/s41467-025-58070-4)
Supplement: Supplementary file 1 — Supplementary Information [file 41467_2025_58070_MOESM1_ESM.pdf]

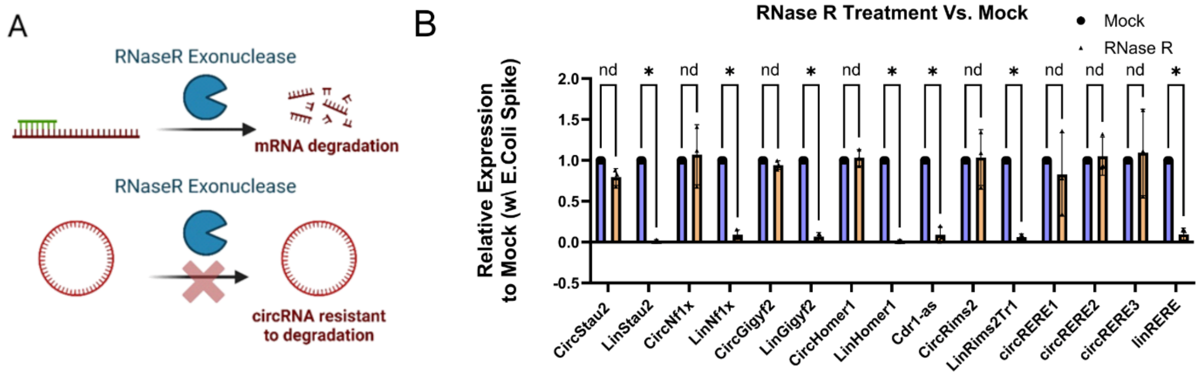

Suppl. Fig 1: Characterisation of the circRNA population in compartmentalised primary rat hippocampal neurons

A. Schematic of Rnase R preferential degradation of linear RNA species, while circular RNAs are more RNase R resistant. B. RNase R treatment of purified RNA from adult rat hippocampus and RT-qPCR of selected circRNA candidates and their linear mRNAs. Cdr1-as is only circRNA displaying notable RNase R sensitivity. N=3 biological replicates. CysG normalization from E.Coli spike-in. Created in BioRender. Schratt, G. (2025) https://BioRender.com/p94x218 . Source data are provided as a Source Data file.

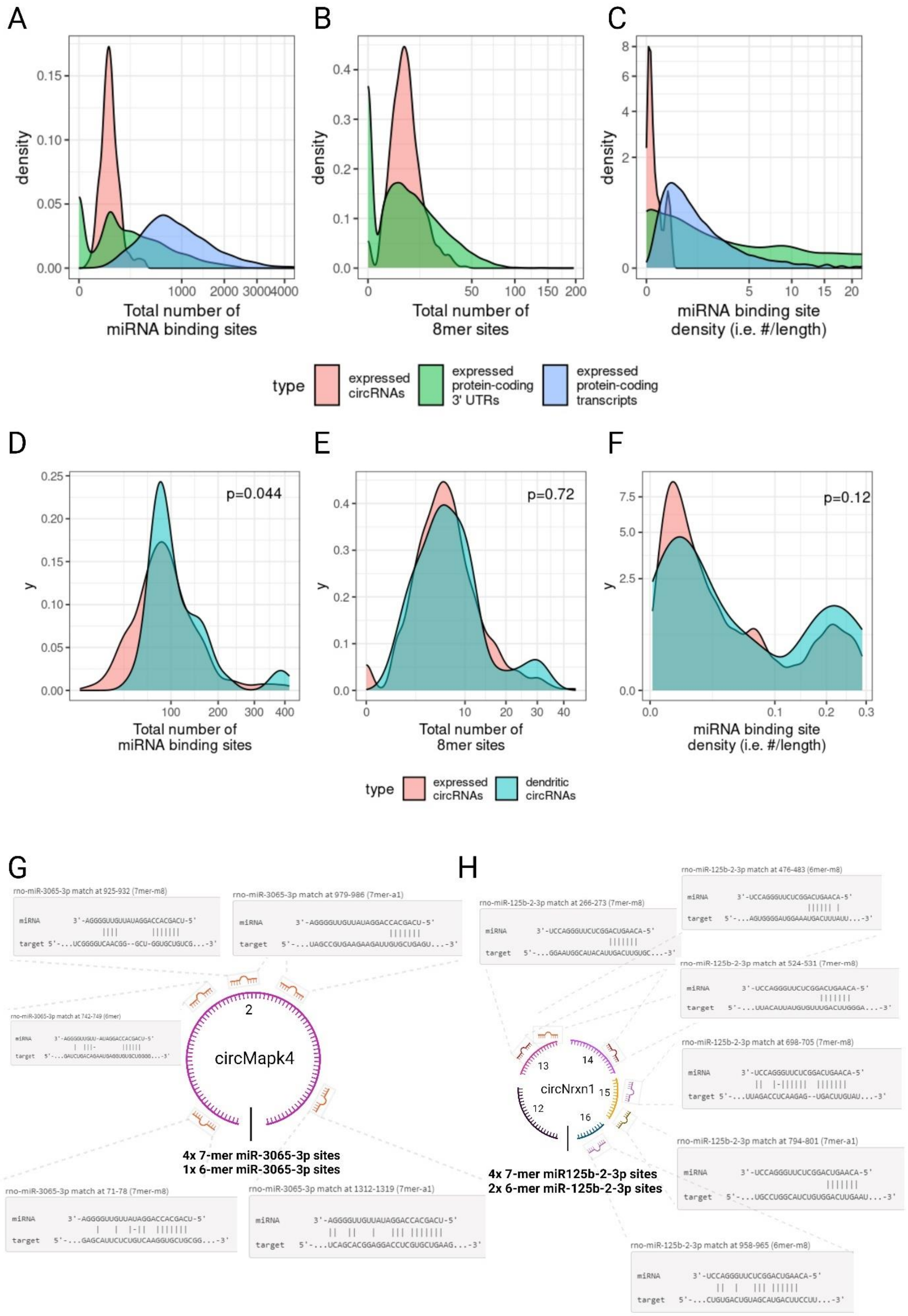
A
B
C
density
Total number of
miRNA binding sites
Total number of
8mer sites
miRNA binding site
density (i.e. #/length)
type
expressed
circRNAs
expressed
protein-coding
3' UTRs
expressed
protein-coding
transcripts
D
E
F
p=0.044
p=0.72
p=0.12
y
Total number of
miRNA binding sites
Total number of
8mer sites
miRNA binding site
density (i.e. #/length)
type
expressed
circRNAs
dendritic
circRNAs
G
H
rno-miR-3065-3p match at 925-932 (7mer-m8)
rno-miR-3065-3p match at 979-986 (7mer-a1)
rno-miR-3065-3p match at 742-749 (6mer)
circMapk4
rno-miR-3065-3p match at 71-78 (7mer-m8)
rno-miR-3065-3p match at 1312-1319 (7mer-a1)
4x 7-mer miR-3065-3p sites
1x 6-mer miR-3065-3p sites
rno-miR-125b-2-3p match at 476-483 (6mer-m8)
rno-miR-125b-2-3p match at 266-273 (7mer-m8)
rno-miR-125b-2-3p match at 524-531 (7mer-m8)
rno-miR-125b-2-3p match at 698-705 (7mer-m8)
circNrxn1
rno-miR-125b-2-3p match at 794-801 (7mer-a1)
rno-miR-125b-2-3p match at 958-965 (6mer-m8)
4x 7-mer miR125b-2-3p sites
2x 6-mer miR-125b-2-3p sites

Suppl. Fig 2: miRNA binding site analysis of circRNA population

Comparison of the total number and density of indicated miRNA site types in Process-enriched circRNAs (red), 3'UTRs of protein-coding mRNAs (green) or entire protein-coding transcripts (blue). A. Indicating the distribution/density of total miRNA binding sites. B. Indicating the distribution/density of 8mer sites. C. Indicating the relative density of miRNA binding sites normalized to species length. Comparison of the total number and density of indicated miRNA site types in all expressed circRNAs (red) and process-enriched circRNAs (blue). D. Indicating the frequency of total number of miRNA binding sites P=0.044, Wilcoxon Test. E. Indicating the frequency of total number of 8mer sites. P=0.72, Wilcoxon Test. F. Indicating the frequency of specific miRNA binding site density. P=0.12, Wilcoxon Test. G. ScanMiR miRNA binding site prediction analysis of dendritically enriched circRNA candidate circMapk4 illustrating 5 predicted miR-3065-3p binding sites. H. ScanMiR analysis of circNrxn1, illustrating 6 predicted miR-125b-2-3p binding sites. Created in BioRender. Schratt, G. (2025) https://BioRender.com/j95u587 . Source data are provided as a Source Data file.

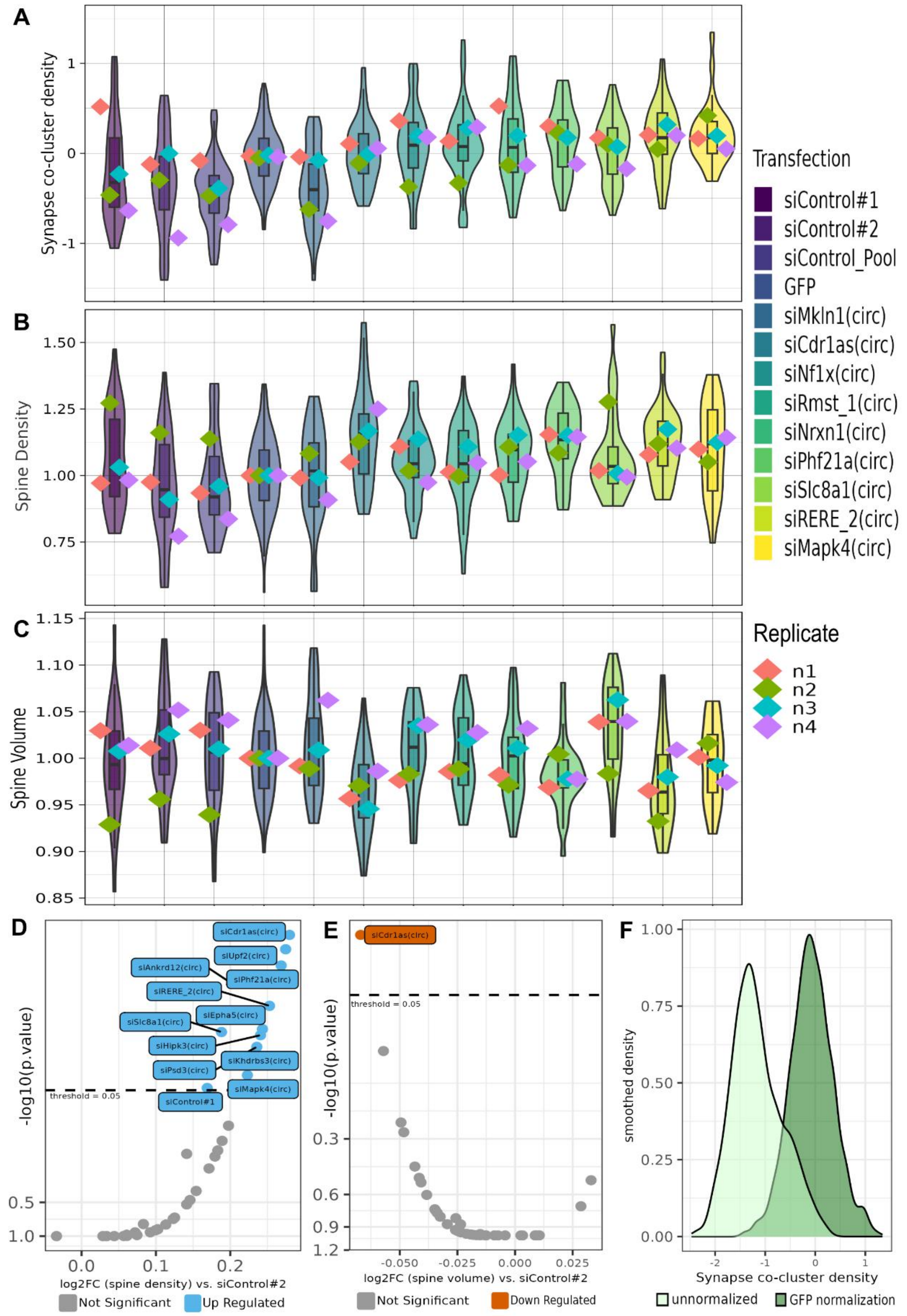
A
Synapse co-cluster density
B
Spine Density
C
Spine Volume
Transfection
siControl#1
siControl#2
siControl_Pool
GFP
siMkln1(circ)
siCdr1as(circ)
siNf1x(circ)
siRmst_1(circ)
siNrxn1(circ)
siPhf21a(circ)
siSlc8a1(circ)
siRERE_2(circ)
siMapk4(circ)
Replicate
n1
n2
n3
n4
D
-log10(p.value)
threshold = 0.05
siCdr1as(circ)
siUpf2(circ)
siAnkrd12(circ)
siPhf21a(circ)
siRERE_2(circ)
siEpha5(circ)
siSlc8a1(circ)
siHipk3(circ)
siKhdrbs3(circ)
siPsd3(circ)
siMapk4(circ)
siControl#1
log2FC (spine density) vs. siControl#2
Not Significant
Up Regulated
E
siCdr1as(circ)
threshold = 0.05
-log10(p.value)
log2FC (spine volume) vs. siControl#2
Not Significant
Down Regulated
F
smoothed density
Synapse co-cluster density
unnormalized
GFP normalization

Suppl. Fig 3: Extended circRNA screen results for synapse co-clusters and dendritic spines.

A. Violin plot related to main Fig. 2C, but showing additional controls (siControl2, siControl_pool). The 7 circRNA siRNAs which demonstrate increased synapse co-cluster density upon knockdown relative to siControl1 also increase relative to siControl2 and siControlPool.

B. Violin of circRNA candidate dendritic spine density upon circRNA knockdown with siRNA pools. siControl1 behaves differently to siControl2 and siControl pool.

C. Volcano plot of circRNA candidate dendritic spine density upon circRNA knockdown with siRNA pools. Depicted are 11/32 circRNA candidates with a significantly increased spine density compared to siControl2 normalised to GFP condition, as determined by GLMM statistical modelling. Note siControl1 significantly different to siControl2.

D. Violin plot of circRNA candidate GFP normalized dendritic spine volume upon circRNA knockdown with siRNA pools.

E. Volcano plot of GFP normalized dendritic spine volume relative to siControl2. Depicted is 1 circRNA candidate, Cdr1-as with a significantly decreased spine volume compared to siControl2 normalised to GFP condition, as determined by GLMM statistical modelling.

F. Smoothed (by standard deviation) density plots showing frequency of synapse co-cluster density between unnormalized and GFP normalized observations. GFP normalized observations are no longer skewed, and better approximate a normal distribution.

For all data N=4 biological replicates, 8 cells per replicate per condition. All statistical comparisons are provided in Supplementary Table 3.

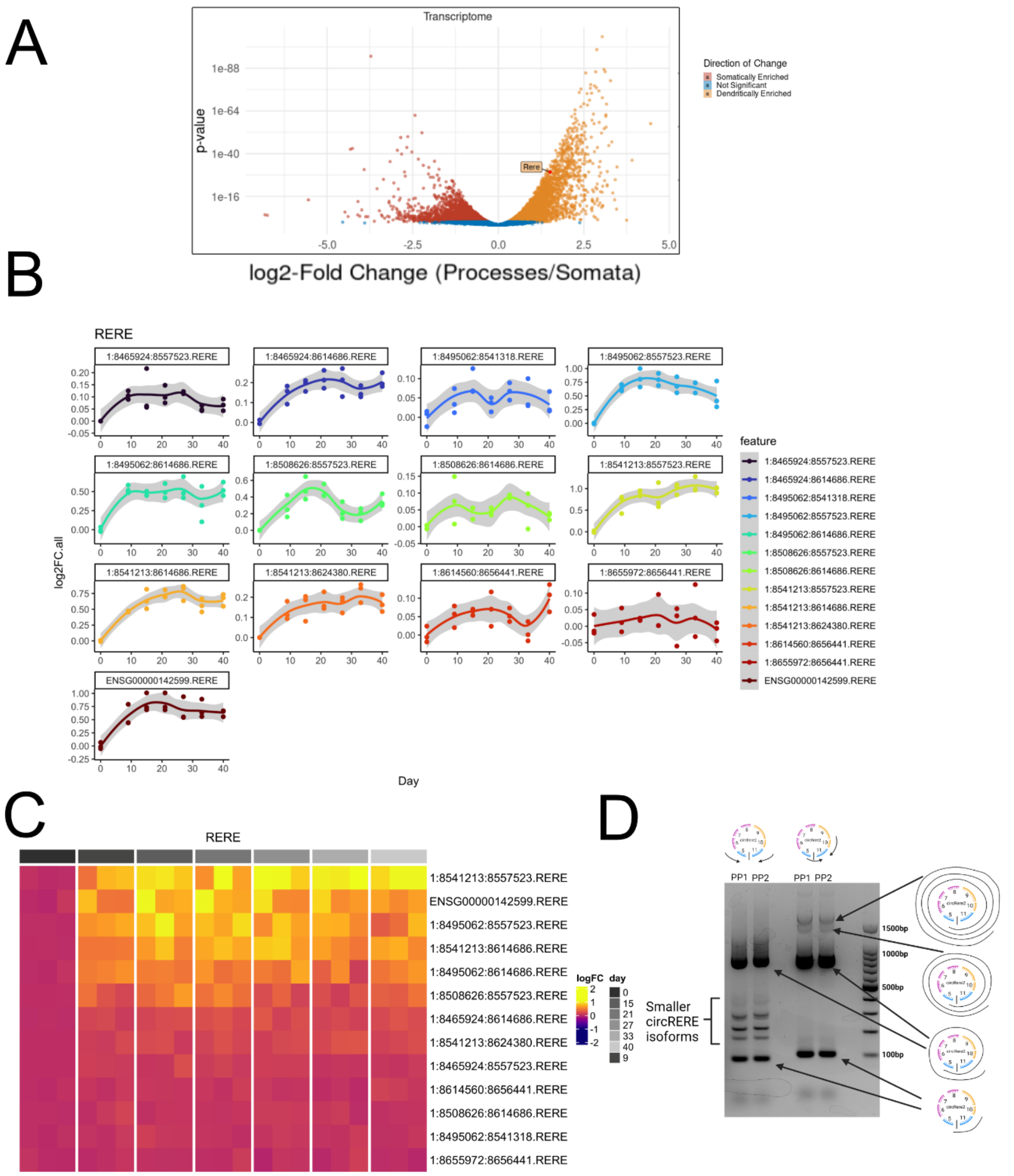

Supp. Fig 4: Extended circRERE expression analysis

A. Volcano plot of all RERE gene reads from Ribo-Minus seq (Colameo et al., 2021), demonstrating overall RERE transcript enrichment in the process compartment. B/C. Timecourse Ribo-Minus sequencing of all circRERE isoforms and linRERE mRNA throughout the development of human iPSCs (Soutschek et al., 2023). D. Rolling circle amplification of circRERE2. Adult rat cortical RNA was subjected to reverse transcription with random hexamers and PCR-amplified using two different pairs of BSJ-flanking or BSJ-spanning divergent qPCR primers (PP1 and PP2). Running PCR samples on TAE 1.5% Agarose gel electrophoresis revealed the expected size for the respective circRNA species. Flanking qPCR primers also amplify smaller circRERE isoforms due to their nested nature and shared exon identity, whereas qPCR with a

BSJ Spanning qPCR primer is specific to circRERE2 alone and demonstrates multiple cycles. N=1. Created in BioRender. Schratt, G. (2025) https://BioRender.com/k29u488 . Source data are provided as a Source Data file (Agarose gel electrophoresis gel).

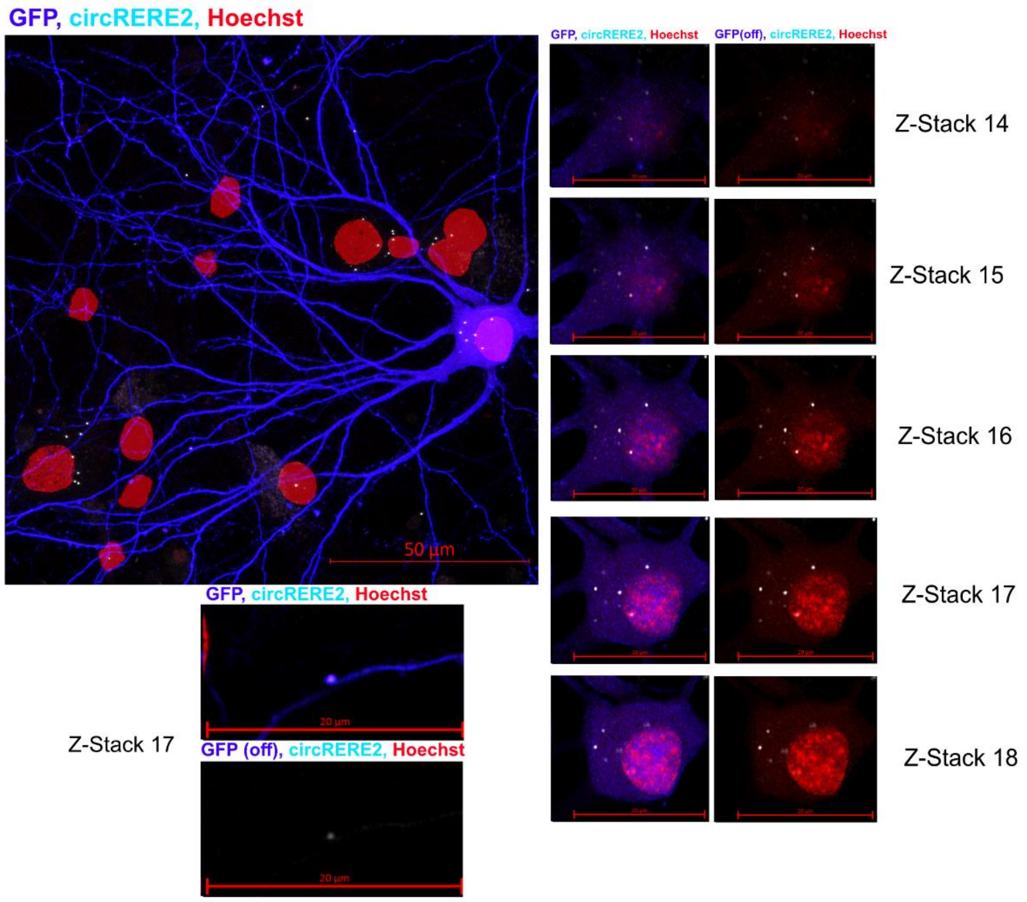

Supp. Fig 5: Z-Stacks of circRNA FISH

Example of airyscan Z-stacks of circRNA FISH confocal microscopy. circRERE2 puncta (white) overlap with GFP signal (blue) in the same confocal stack (Z-stack). circRERE2 signal in the soma overlaps with nucleus in some cases, but circRERE2 signal and Hoechst signal (red) do not consistently overlap, e.g. Z-stack 14/15. Soma signal is not inside nucleus. circRERE2 signal is also detected in dendritic process/spines at same Z-Stack.

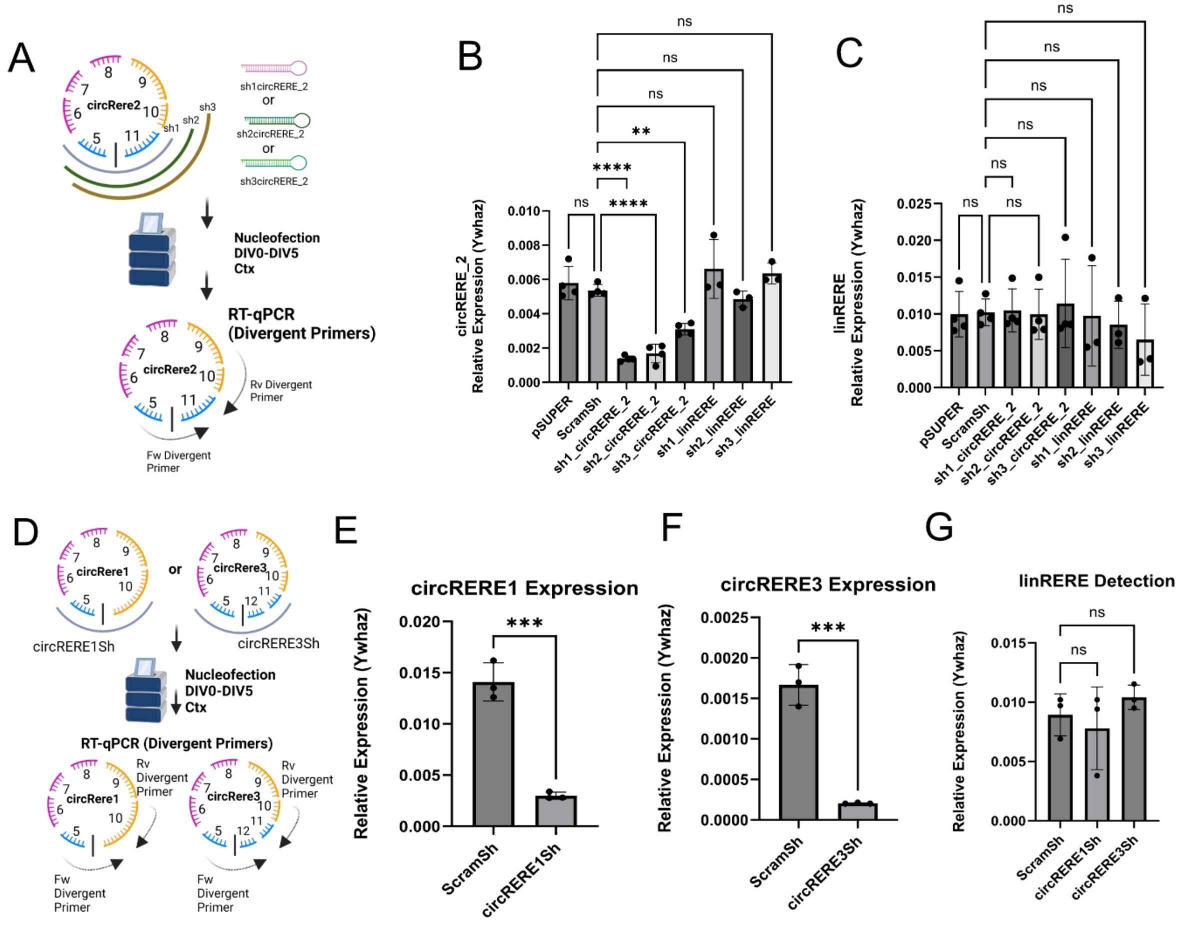

Supp. Fig 6: Validation of circRERE isoform knockdown efficiency/specificity

A/D. Schematics for the shRNA and primer design for circRERE2 RT-qPCR validation and circRERE1/3 respectively. B/E/F. Relative expression levels of indicated circRERE isoforms upon nucleofection/electroporation of the respective shRNA constructs as determined by qPCR using BSJ spanning divergent primers, Ywhaz normalization. C/G. Relative expression levels of linear RERE upon nucleofection of the respective shRNA constructs as determined by qPCR. N=3-4, P<0.05 *, p<0.01** P<0.001 ***, P<0.0001 ****. Created in BioRender. Schratt, G. (2025) https://BioRender.com/r32x446 . Source data are provided as a Source Data file, including exact p-values and details of statistical tests.

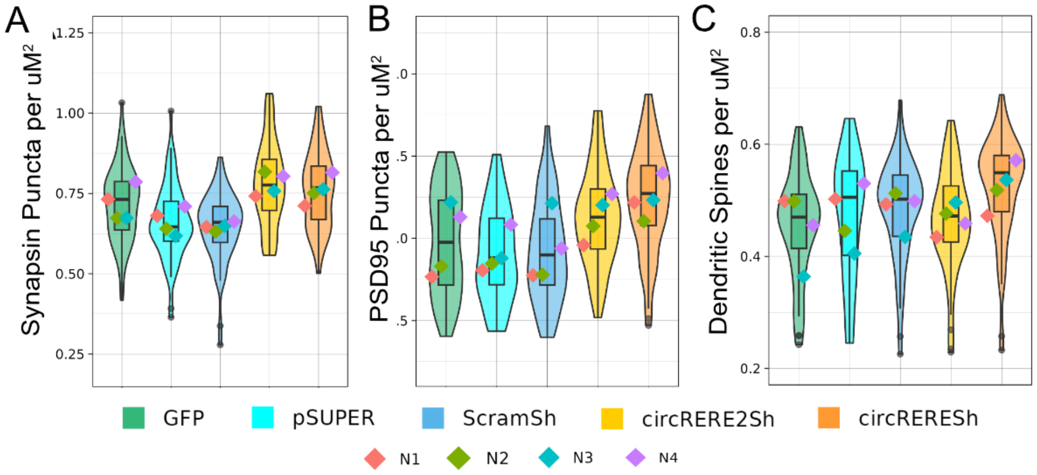

Supp. Fig 7: Extended circRERE shRNA results for synapse co-clusters and dendritic spines

Violin plots related to main Fig. 3G, illustrating A. Synapsin Puncta per uM^2, B. PSD95 Puncta per uM^2 and C. Dendritic Spines per uM^2, for the labelled conditions. Statistical comparisons and information as determined by GLMM statistical modelling as per Fig. 3G (Source data) are provided as a Source Data file, including exact p-values and details of statistical tests.

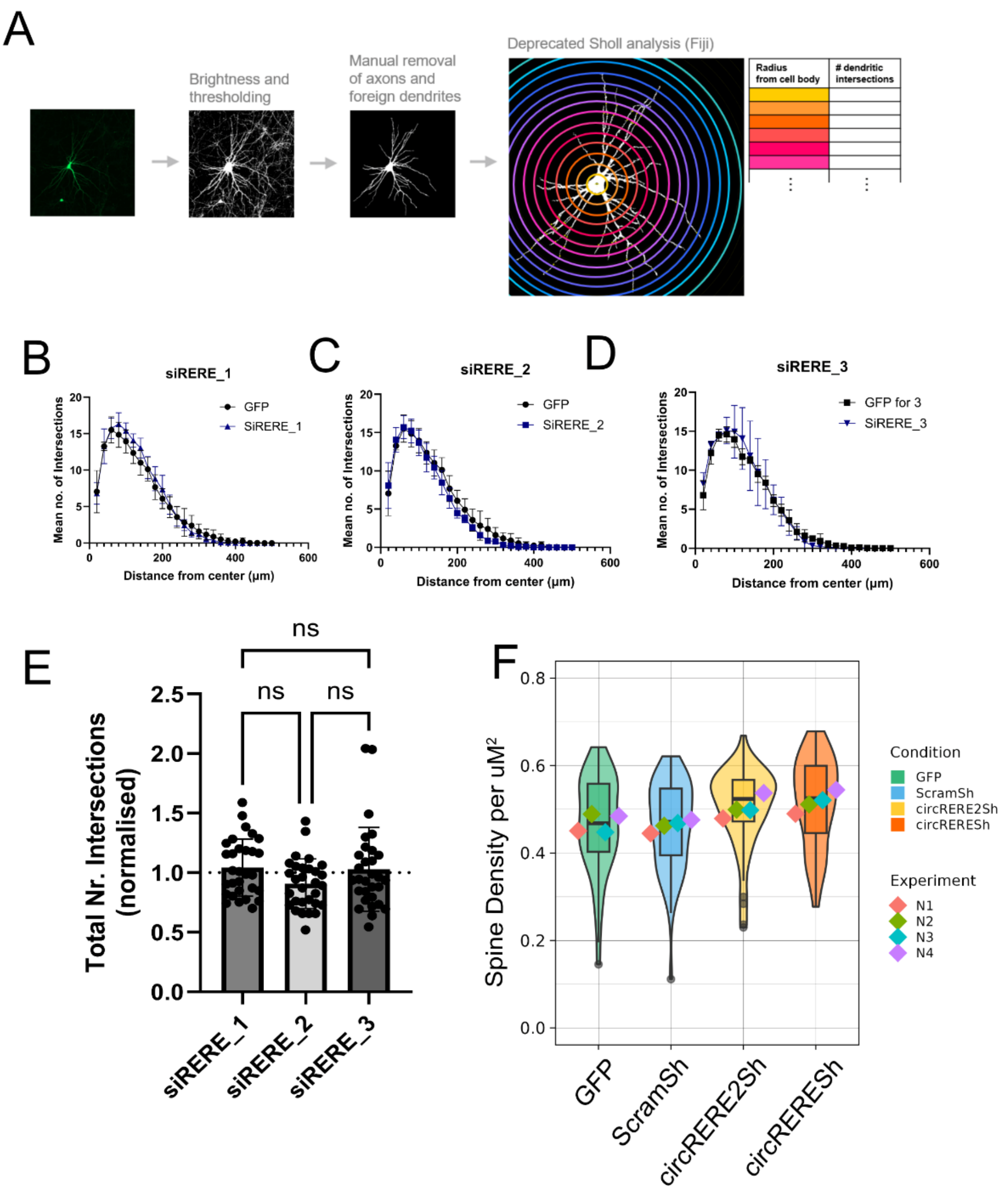

Supp. Fig 8: Extended morphological analysis of circRERE kd neurons.

A. Illustration of the Sholl analysis pipeline used for the analysis of dendritogenesis. B-D: Mean number of intersections in rat hippocampal neurons (DIV12) transfected with the indicated siRNAs (red) or control GFP plasmid (black). 1-way ANOVA, Sidak Multiple Comparison test. No significant changes, siRERE_2 (P<0.3592). E. Mean number of total intersections for conditions described on the left. N=3 biological replicates, 9-10 Cells per conditions, per replicate, normalised to GFP Control conditions. 1-way ANOVA, Sidak Multiple Comparison test. No significant changes Mean intersections (P<0.3822). F. Violin plot relating to main Fig. 4K, illustrating Dendritic Spines per uM^2, GLMM statistical modelling, comparisons (source data) are provided as a Source Data file, including exact p-values, and details of statistical tests.

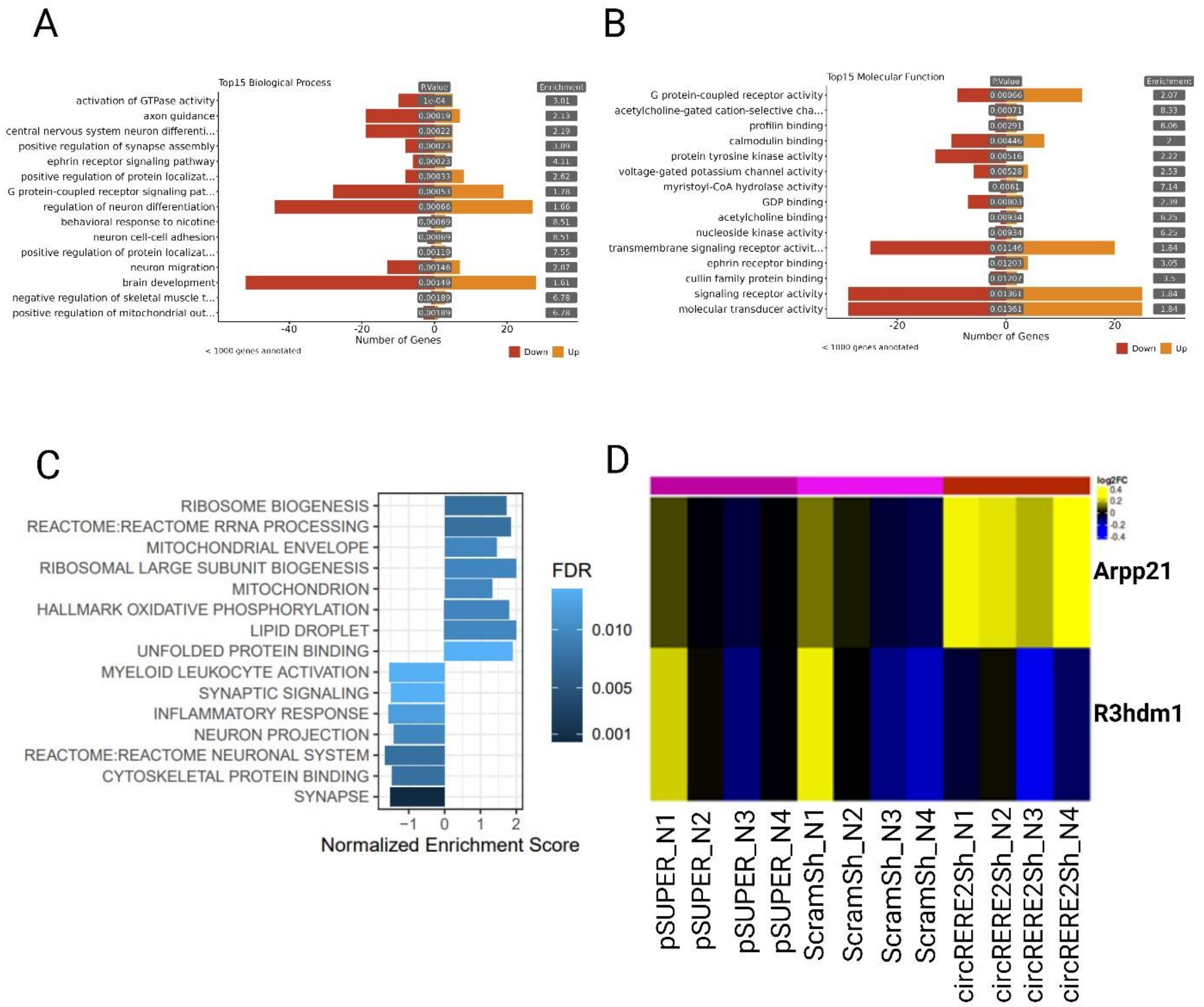

Supp. Fig 9: circRERE2kd Poly-A seq additional characterisation

A. Biological Process and B. Molecular Function GO Term analysis (topGO) of DEGs (circRERE2Sh vs.ScramSh/pSUPER Controls). C. Gene-set enrichment analysis in (circRERE2Sh vs. ScramSh/pSUPER Controls). D. Heatmap of miR-128-3p host genes (Arpp21, R3hdm1) upon circRERE2Sh. Arpp21 expression is (non-significantly) (FDR<0.0674) increased (circRERE2Sh vs.ScramSh/pSUPER Controls). Source data are provided as a Source Data file.

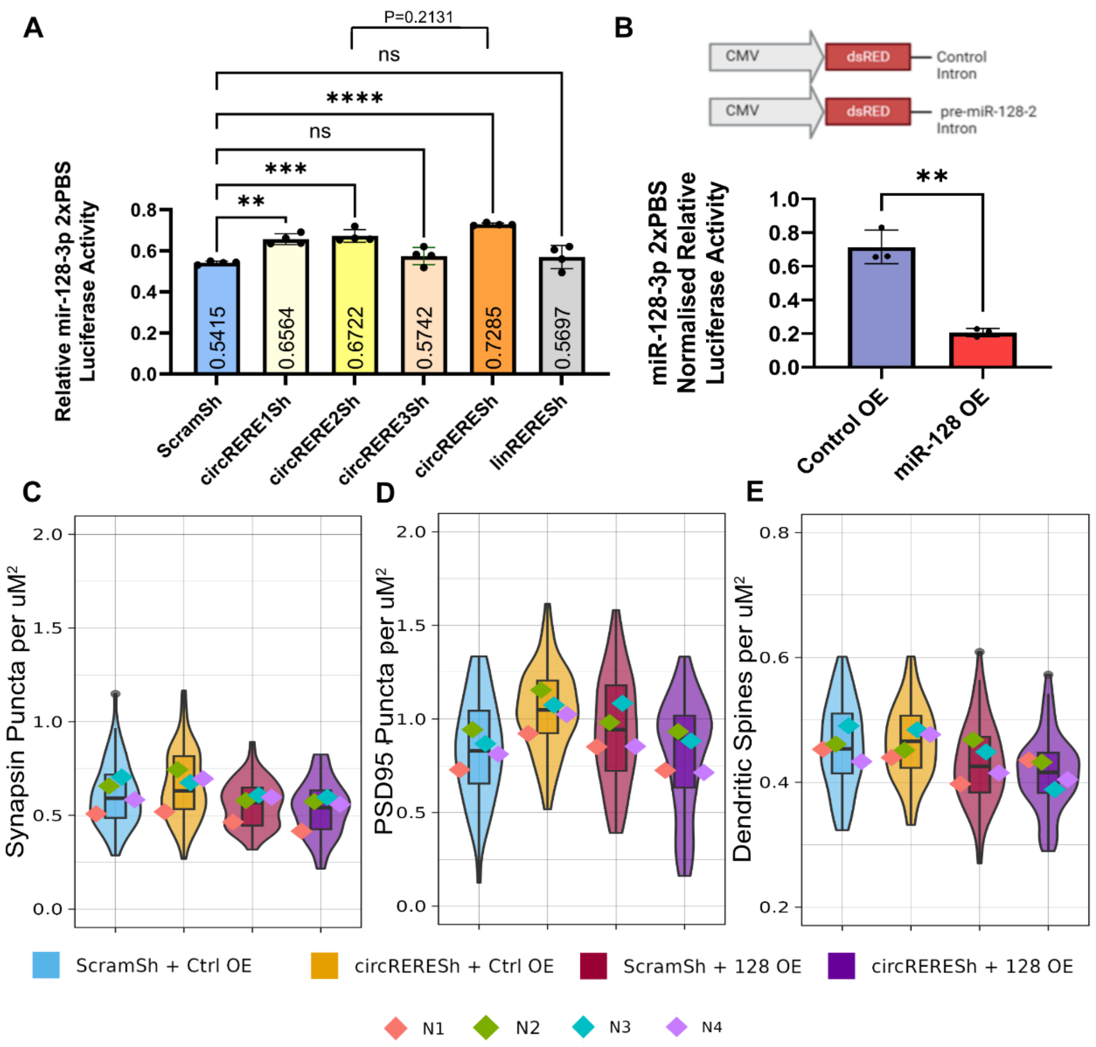

Supp. Fig 10: Extended circRERE isoform, miR-128-OE luciferase assays and miR-128OE morphological characterization

A. Relative luciferase activity in rat cortical neurons transfected with miR-128-3p 2x PBS together with the indicated shRNA constructs. N=4, unpaired t-test, P<0.05, Source data and statistical information are provided as a Source Data file. B. Upper panel: Illustration of dsRED-pre-miR-128-2 overexpression construct and control. Lower panel: Relative luciferase activity in neurons transfected with miR-128-3p 2x PBS together with the indicated miR-128-3p overexpression (OE) constructs. N=3. Unpaired student's t-test P<0.01 **. Created in BioRender. Schratt, G. (2025) https://BioRender.com/a87e574 . Source data and statistical information are provided as a Source Data file. C-E. Violin plots related to main Fig. 6H, illustrating A. Synapsin Puncta per uM^2, B. PSD95 Puncta per uM^2 and C. Dendritic Spines per uM^2, for the labelled conditions. Statistical comparisons and information as determined by GLMM statistical modelling as per Fig. 6H, Source data and additional statistical information are provided as a Source Data file.

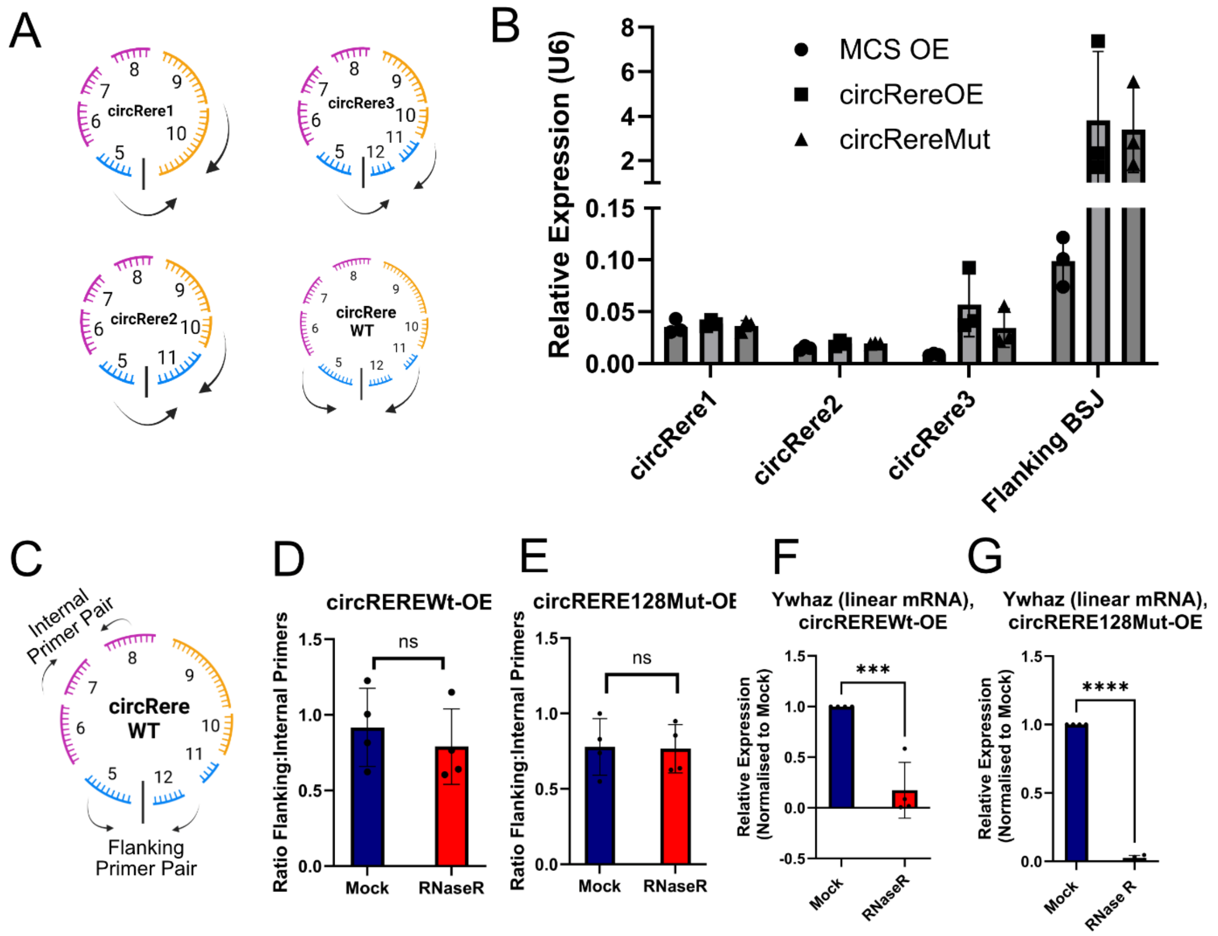

Supp. Fig 11: circRERE Overexpression Validations for efficacy and circularity

A/B: Relative expression of indicated circRERE isoforms in primary cortical neurons electroporated with the indicated circRERE or control OE constructs as determined by qPCR. circRERE isoform structure and qPCR primer design is shown on the right. N=3 independent biological replicates, U6 Normalization N=3. Source data and statistical information are provided as a Source Data file. C-G: circRERE-OE validation of circularity by comparison of circREREOE BSJ flanking primers (circular specific) and internal primers (linear and circular) as visualised in schematic C. In both circREREWT-OE (D/F) and circRERE128Mut-OE (E/G), the ratio of Flanking:Internal primers in not significantly different upon RNase R treatment. However, the Ywhaz mRNA positive control demonstrates efficient sensitivity to RNase R treatment. N=4, P<0.001 ***, P<0.0001 ****. Created in BioRender. Schratt, G. (2025) https://BioRender.com/f73a051 . Source data and statistical information are provided as a Source Data file.

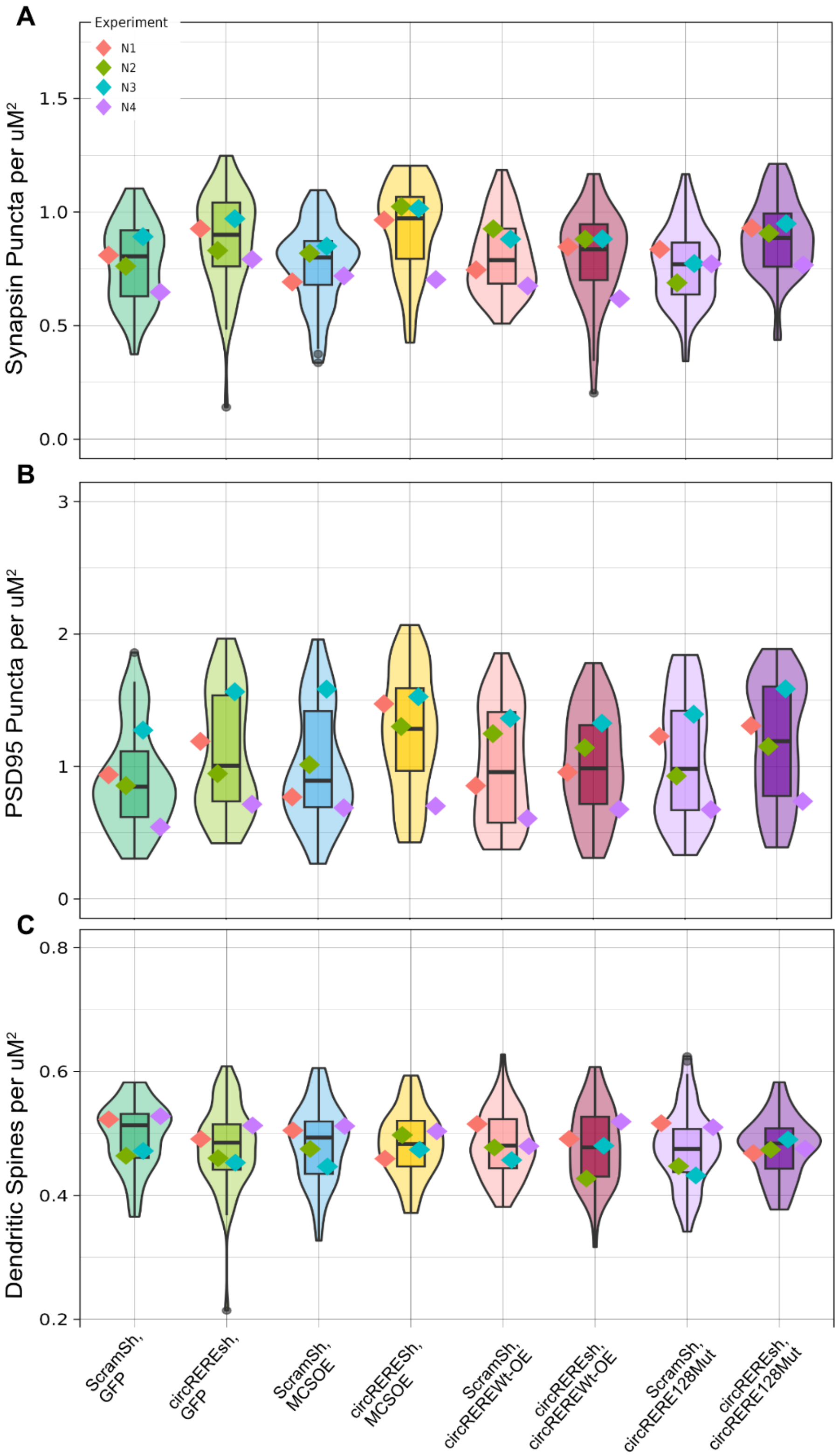
A
Experiment
N1
N2
N3
N4
Synapsin Puncta per uM²
1.5
1.0
0.5
0.0
B
PSD95 Puncta per uM²
3
2
1
0
C
Dendritic Spines per uM²
0.8
0.6
0.4
0.2
ScramSh, GFP
circREREsh, GFP
ScramSh, MCSOE
circREREsh, MCSOE
ScramSh, circREREWt-OE
circREREsh, circREREWt-OE
ScramSh, circRERE128Mut
circREREsh, circRERE128Mut

Supp. Fig 12: Extended circREREOE results for synapse co-clusters and dendritic spines

Violin plots related to main Fig. 7D, illustrating A. Synapsin Puncta per uM^2, B. PSD95 Puncta per uM^2 and C. Dendritic Spines per uM^2, for the labelled conditions. Statistical comparisons and information as determined by GLMM statistical modelling as per Fig. 7D, Source data and additional statistical information are provided as a Source Data file.

Supplementary Primer List :

qPCR primers (Rattus Norvegicus)

GAPDH fw: GCCTTCTCTTGTGACAAAGTGGA

GAPDH rv: CCGTGGGTAGAGTCATACTGGAA

Ywhaz Fw: CTCCGGACACAGAATATCCAGT

Ywhaz Rv: TGCCATGTCATCGTATCGCT

U6 snRNA Fw: CTCGCTTCGGCAGCACA

U6 snRNA Rv: AACGCTTCACGAATTTGCGT

Cdr1-as (Circular) Fw: CTGCCGTATCCAGGGGTTT

Cdr1-as (Circular) Rv: TGGAAGACCTTGACAGTGTTGG

circRims2 Fw: CTCTCACGGAAAAGTCGCAGT

circRims2 Rv: AGAGGCCGTTGTTCTGTTGATC

linRims2Tr1 Fw: GCGTAGGTGAGAAAGGAGACA

linRims2Tr1 Rv: CTGCCAGGTCACAGGGT

circHomer1 Fw: TGCCATTTTCACATAGGGAAC

circHomer1 Rv: TAACTGCATGCTTGCTGGTG

circGigyf2 Fw: AGGAAGAAAAGATGTAGGCTCCG

circGigyf2 Rv: TCGGCCATATCGATAATCTGC

linGigyf2 Fw: ACAGCCTGCAGTTTGGGAA

linGigyf2 Rv: AGCTTTGGCCTTTTCCAGC

circNf1x Fw: GACCTTTATCTGGCTTACTTTGTCC

circNf1x Rv: GAACCAGGTGTAGGAGAAGGC

linNf1x Fw: CACACCCAACCATCCGCTAC

linNf1x Rv: TTGTGAATGCTGCCCGGT

circStau2 Fw: CAACTTCCGGGGCATGTA

circStau2 Rv: GGGAGACCTGGAGAGAAGCTG

linStau2 Fw: TGAAGTTGCGACTGGAACAG

linStau2 Rv: TGATCCTGAAGACTAGTGGA

circSlc8a1 Fw: CCAGAATGATGAAATAGTGTTGGAAC

circSlc8a1 Rv: CCACCAGAGTTACCAGACGAAAT

circAnks1b: Fw GGAAGCCAGAGTGTAACAGAGAAG

circAnks1b: Rv CCATAGAGAATCATGATAGCTACCTGT

circSnap25 Fw: GACACCCAGAATCGCCAGA

circSnap25 Rv: CCAGCATCTTTGTTGCACG

circPsd3 Fw: CTCCAAGGATCTTCTAAAATAGTGGC

circPsd3 Rv: CTCTGTGGTCTCCTTTTCCAGAA

circAnks1b Fw: GGAAGCCAGAGTGTAAACAACG

circAnks1b Rv: GGGATCGGTGAGCTCCTCTA

circNckap1 Fw: TTAACGAGCTTGTGCAGAATCTCT

circNckap1 Rv: GCTTTCTTACCTTGTTTCACACTTAGG

CysG Fw E.Coli: TTGTCGGCGGTGGTGATGTC

CysG Rv E.Coli: ATGCGGTGAACTGTGGAATAAACG

circRERE2 Flanking (2 sets, Rolling Circle Amp)

circRERE2 Flanking fw1: GTGCCCAAGCTGATCGAGAA

circRERE2 Flanking Rv1: CTGACGGTAGTACCATTTGACG

circRERE2 Flanking Fw2: TCGAGAAGTGCTGGACAGAG

circRERE2 Flanking Rv2: CTGGAACTTCAGACTGACGGTAG

circRERE2 BSJ spanning (qPCR)

circRERE2 BSJ spanning Fw: TGGACAGAGGATGAAGTGAGT

circRERE2 BSJ spanning Rv: GGTCCTGAACCAAATGCTGA

circRERE1 BSJ Spanning (qPCR)

circRERE1 BSJ Spanning Fw: GCACTGAACACAAGTAAGAGG

circRERE1 BSJ Spanning Rv: TGCCGGTCCTGAACCAAATG

circRERE3 BSJ Spanning (qPCR)

circRERE3 BSJ Spanning Fw: AGGAAACCAGTAAGAGGGACCA

circRERE3 BSJ Spanning Rv: ATGCCGGTCCTGAACCAAAT

circREREWt Internal: (qPCR)

Fw GGGAAGTGCAACATCTCCCA (Exon 7)

Rv GCCTCCTTGTCTCAGGGTTG (Exon 7-8)

circRERE Flanking: (qPCR)

Fw GAAACGCTTCGTTAAGGGGC (Exon 11-12):

Rv ATGCCGGTCCTGAAcCAAAT (Exon 5)

linRERE (qPCR)

linRERE (Exons 21) Fw: CCTCACTTAGCTCGCTTCCC

linRERE (Exons 22-21) Rv: GGTAGGGGGTGCCAAAAACT

siRNAs:

**Target RNA Sequence / siRNA ID**	**Primer Name**	**sense**	**antisense**
ACUUAAAGUGUUCUAAGAA	**Whsc1 #1**	**ACUUAAAGUGUUCUAAGAATT**	**UUCUUAGAACACUUUAAGUTT**
AAACUUAAAGUGUUCUAAG	**Whsc1 #2**	**AAACUUAAAGUGUUCUAAGTT**	**CUUAGAACACUUUAAGUUUTT**
CCAAACUUAAAGUGUUCUA	**Whsc1 #3**	**CCAAACUUAAAGUGUUCUATT**	**UAGAACACUUUAAGUUUGGTT**
GGUAAAUGGAACAUUUUAG	**Mkln1 #1**	**GGUAAAUGGAACAUUUUAGTT**	**CUAAAAUGUUCCAUUUACCTT**
GCGGUAAAUGGAACAUUUU	**Mkln1 #2**	**GCGGUAAAUGGAACAUUUUTT**	**AAAAUGUUCCAUUUACCGCTT**
AAGCGGUAAAUGGAACAUU	**Mkln1 #3**	**AAGCGGUAAAUGGAACAUUTT**	**AAUGUUCCAUUUACCGCUUTT**
GAAAUAGUGUUGGAACAAU	**Slc8a1 #1**	**GAAAUAGUGUUGGAACAAUTT**	**AUUGUUCCAACACUAUUUCTT**
AUGAAAUAGUGUUGGAACA	**Slc8a1 #2**	**AUGAAAUAGUGUUGGAACATT**	**UGUUCCAACACUAUUUCAUTT**
UGAUGAAAUAGUGUUGGAA	**Slc8a1 #3**	**UGAUGAAAUAGUGUUGGAATT**	**UUCCAACACUAUUUCAUCATT**
UUAUACAGAUCCAGGAUGA	**Ankrd12 #1**	**UUAUACAGAUCCAGGAUGATT**	**UCAUCCUGGAUCUGUAUAATT**
AGUUAUACAGAUCCAGGAU	**Ankrd12 #2**	**AGUUAUACAGAUCCAGGAUTT**	**AUCCUGGAUCUGUAUAACUTT**
AAAGUUAUACAGAUCCAGG	**Ankrd12 #3**	**AAAGUUAUACAGAUCCAGGTT**	**CCUGGAUCUGUAUAACUUUTT**
CAAUACAGUGCAAUGGCAA	**Nrcam #1**	**CAAUACAGUGCAAUGGCAATT**	**UUGCCAUUGCACUGUAUUGTT**

ACAAUACAGUGCAAUGGCA	**Nrcam #2**	**ACAAUACAGUGCAAUGGCATT**	**UGCCAUUGCACUGUAUUGUTT**
AUACAGUGCAAUGGCAAGU	**Nrcam #3**	**AUACAGUGCAAUGGCAAGUTT**	**ACUUGCCAUUGCACUGUAUTT**
CAAUGACCGCAAAGGUCCU	**Nrxn1 #1**	**CAAUGACCGCAAAGGUCCUTT**	**AGGACCUUUGCGGUCAUUGTT**
UGCAAUGACCGCAAAGGUC	**Nrxn1 #2**	**UGCAAUGACCGCAAAGGUCTT**	**GACCUUUGCGGUCAUUGCATT**
UCUGCAAUGACCGCAAAGG	**Nrxn1 #3**	**UCUGCAAUGACCGCAAAGGTT**	**CCUUUGCGGUCAUUGCAGATT**
UGUCAAGUGGGAAGAGAUU	**Epha5 #1**	**UGUCAAGUGGGAAGAGAUUTT**	**AAUCUCUUCCCACUUGACATT**
CUGUCAAGUGGGAAGAGAU	**Epha5 #2**	**CUGUCAAGUGGGAAGAGAUTT**	**AUCUCUUCCCACUUGACAGTT**
CCUGUCAAGUGGGAAGAGA	**Epha5 #3**	**CCUGUCAAGUGGGAAGAGATT**	**UCUCUUCCCACUUGACAGGTT**
CCAAAGAUACUCAUGCUAA	**Rmst_1 #1**	**CCAAAGAUACUCAUGCUAATT**	**UUAGCAUGAGUAUCUUUGGTT**
GCCAAAGAUACUCAUGCUA	**Rmst_1 #2**	**GCCAAAGAUACUCAUGCUATT**	**UAGCAUGAGUAUCUUUGGCTT**
AGCCAAAGAUACUCAUGCU	**Rmst_1 #3**	**AGCCAAAGAUACUCAUGCUTT**	**AGCAUGAGUAUCUUUGGCUTT**
CACACUCCGGGAUGAGUUC	**Nf1x #1**	**CACACUCCGGGAUGAGUUCTT**	**GAACUCAUCCCGGAGUGUGTT**
CUCCGGGAUGAGUUCCACC	**Nf1x #2**	**CUCCGGGAUGAGUUCCACCTT**	**GGUGGAACUCAUCCCGGAGTT**
UCCACACUCCGGGAUGAGU	**Nf1x #3**	**UCCACACUCCGGGAUGAGUTT**	**ACUCAUCCCGGAGUGUGGATT**
UGAACACAAGUAAGAGGGA	**RERE_1 #1**	**UGAACACAAGUAAGAGGGATT**	**UCCCUCUUACUUGUGUUCATT**
GAACACAAGUAAGAGGGAC	**RERE_1 #2**	**GAACACAAGUAAGAGGGACTT**	**GUCCCUCUUACUUGUGUUCTT**
AACACAAGUAAGAGGGACC	**RERE_1 #3**	**AACACAAGUAAGAGGGACCTT**	**GGUCCCUCUUACUUGUGUUTT**
CCAGAGUGUAACAGAGAAG	**Anks1b_1 #1**	**CCAGAGUGUAACAGAGAAGTT**	**CUUCUCUGUUACACUCUGGTT**
GCCAGAGUGUAACAGAGAA	**Anks1b_1 #2**	**GCCAGAGUGUAACAGAGAATT**	**UUCUCUGUUACACUCUGGCTT**
AGUGUAACAGAGAAGGGGA	**Anks1b_1 #3**	**AGUGUAACAGAGAAGGGGATT**	**UCCCCUUCUCUGUUACACUTT**
AGAGUGUAAACAACGAGAA	**Anks1b_2 #1**	**AGAGUGUAAACAACGAGAATT**	**UUCUCGUUGUUUACACUCUTT**
GCCAGAGUGUAAACAACGA	**Anks1b_2 #2**	**GCCAGAGUGUAAACAACGATT**	**UCGUUGUUUACACUCUGGCTT**
GAGUGUAAACAACGAGAAC	**Anks1b_2 #3**	**GAGUGUAAACAACGAGAACTT**	**GUUCUCGUUGUUUACACUCTT**
AUGAAGUGAGUAAGAGGGA	**RERE_2 #1**	**AUGAAGUGAGUAAGAGGGATT**	**UCCCUCUUACUCACUUCAUTT**
GAAGUGAGUAAGAGGGACC	**RERE_2 #2**	**GAAGUGAGUAAGAGGGACCTT**	**GGUCCCUCUUACUCACUUCTT**

GGAUGAAGUGAGU AAGAGG	**RERE_2 #3**	**GGAUGAAGUGAGU AAGAGGTT**	**CCUCUUACUCACU UCAUCCTT**
GGCAAGAAAUAUA GGAGUA	**Foxn2 #1**	**GGCAAGAAAUAUA GGAGUATT**	**UACUCCUAUAUUU CUUGCCTT**
CGGCAAGAAAUAU AGGAGU	**Foxn2 #2**	**CGGCAAGAAAUAU AGGAGUTT**	**ACUCCUAUAUUUC UUGCCGTT**
ACGGCAAGAAAUA UAGGAG	**Foxn2 #3**	**ACGGCAAGAAAUA UAGGAGTT**	**CUCCUAUAUUUCU UGCCGUTT**
UUUCACAUAGGGA ACAACC	**Homer1 #1**	**UUUCACAUAGGGA ACAACCTT**	**GGUUGUUCCCUAU GUGAAATT**
CCAUUUUCACAUA GGGAAC	**Homer1 #2**	**CCAUUUUCACAUA GGGAACTT**	**GUUCCCUAUGUGA AAAUGGTT**
UCACAUAGGGAAC AACCUA	**Homer1 #3**	**UCACAUAGGGAAC AACCUATT**	**UAGGUUGUUCCCU AUGUGATT**
UUGCUGCGAAUUU UAUAGG	**Uvrag #1**	**UUGCUGCGAAUUU UAUAGGTT**	**CCUAUAAAAUUCG CAGCAATT**
CUUUGCUGCGAAU UUUAUA	**Uvrag #2**	**CUUUGCUGCGAAU UUUAUATT**	**UAUAAAAUUCGCA GCAAAGTT**
UUCUUUGCUGCGA AUUUUA	**Uvrag #3**	**UUCUUUGCUGCGA AUUUUATT**	**UAAAAUUCGCAGC AAAGAATT**
AUGGAGAGUAUUU CAACUU	**Khdrbs3 #1**	**AUGGAGAGUAUUU CAACUUTT**	**AAGUUGAAAUACU CUCCAUTT**
GGAGAGUAUUUCA ACUUUG	**Khdrbs3 #2**	**GGAGAGUAUUUCA ACUUUGTT**	**CAAAGUUGAAAUA CUCUCCTT**
GAGAGUAUUUCAA CUUUGU	**Khdrbs3 #3**	**GAGAGUAUUUCAA CUUUGUTT**	**ACAAAGUUGAAAU ACUCUCTT**
GCCUGUGUGGGA AUAGCUA	**Zranb1 #1**	**GCCUGUGUGGGAA UAGCUATT**	**UAGCUAUUCCCAC ACAGGCTT**
CCUGUGUGGGAAU AGCUAU	**Zranb1 #2**	**CCUGUGUGGGAAU AGCUAUTT**	**AUAGCUAUUCCCA CACAGGTT**
AUGCCUGUGUGG GAAUAGC	**Zranb1 #3**	**AUGCCUGUGUGGG AAUAGCTT**	**GCUAUUCCCACAC AGGCAUTT**
UGUGCAAAAAGAA ACUUGU	**Ubn2 #1**	**UGUGCAAAAAGAA ACUUGUTT**	**ACAAGUUUCUUUU UGCACATT**
UCUGUGCAAAAAG AAACUU	**Ubn2 #2**	**UCUGUGCAAAAAG AAACUUTT**	**AAGUUUCUUUUUG CACAGATT**
CUUCUGUGCAAAA AGAAAC	**Ubn2 #3**	**CUUCUGUGCAAAA AGAAACTT**	**GUUUCUUUUUGCA CAGAAGTT**
AUGGCUUCAUGAG AUACUC	**Rmst_2 #1**	**AUGGCUUCAUGAG AUACUCTT**	**GAGUAUCUCAUGA AGCCAUTT**
GGCUUCAUGAGAU ACUCAU	**Rmst_2 #2**	**GGCUUCAUGAGAU ACUCAUTT**	**AUGAGUAUCUCAU GAAGCCTT**
UCAUGAGAUACUC AUGCUA	**Rmst_2 #3**	**UCAUGAGAUACUC AUGCUATT**	**UAGCAUGAGUAUC UCAUGATT**
AGGAAACCAGUAA GAGGGA	**RERE_3 #1**	**AGGAAACCAGUAA GAGGGATT**	**UCCCUCUUACUGG UUUCCUTT**
GAAACCAGUAAGA GGGACC	**RERE_3 #2**	**GAAACCAGUAAGA GGGACCTT**	**GGUCCCUCUUACU GGUUUCTT**
UAAGGAAACCAGU AAGAGG	**RERE_3 #3**	**UAAGGAAACCAGU AAGAGGTT**	**CCUCUUACUGGUU UCCUUATT**

AUGGCAAGGUAGGUGAGCC	**Mapkap1 #1**	**AUGGCAAGGUAGGUGAGCCTT**	**GGCUCACCUACCUUGCCAUTT**
UGAUGGCAAGGUAGGUGAG	**Mapkap1 #2**	**UGAUGGCAAGGUAGGUGAGTT**	**CUCACCUACCUUGCCAUCATT**
UUUGAUGGCAAGGUAGGUG	**Mapkap1 #3**	**UUUGAUGGCAAGGUAGGUGTT**	**CACCUACCUUGCCAUCAAATT**
UCAUGUUGGUUCCCAGUCA	**Ash1l #1**	**UCAUGUUGGUUCCCAGUCATT**	**UGACUGGGAACCAACAUGATT**
CAUUCAUGUUGGUUCCCAG	**Ash1l #2**	**CAUUCAUGUUGGUUCCCAGTT**	**CUGGGAACCAACAUGAAUGTT**
AUUCAUGUUGGUUCCCAGU	**Ash1l #3**	**AUUCAUGUUGGUUCCCAGUTT**	**ACUGGGAACCAACAUGAAUTT**
AGAAACAGGGGACUAGAAA	**Phf21a #1**	**AGAAACAGGGGACUAGAAATT**	**UUUCUAGUCCCCUGUUUCUTT**
GAGAAACAGGGGACUAGAA	**Phf21a #2**	**GAGAAACAGGGGACUAGAATT**	**UUCUAGUCCCCUGUUUCUCTT**
AAACAGGGGACUAGAAAGC	**Phf21a #3**	**AAACAGGGGACUAGAAAGCTT**	**GCUUUCUAGUCCCCUGUUUTT**
AGGCAGUGUGAAAACUCAG	**Ralgapa1 #1**	**AGGCAGUGUGAAAACUCAGTT**	**CUGAGUUUUCACACUGCCUTT**
CAAGGCAGUGUGAAAACUC	**Ralgapa1 #2**	**CAAGGCAGUGUGAAAACUCTT**	**GAGUUUUCACACUGCCUUGTT**
UUCAAGGCAGUGUGAAAAC	**Ralgapa1 #3**	**UUCAAGGCAGUGUGAAAACTT**	**GUUUUCACACUGCCUUGAATT**
CCACAAGGGAAGGGGACUG	**Mapk4 #1**	**CCACAAGGGAAGGGGACUGTT**	**CAGUCCCCUUCCCUUGUGGTT**
CCCACAAGGGAAGGGGACU	**Mapk4 #2**	**CCCACAAGGGAAGGGGACUTT**	**AGUCCCCUUCCCUUGUGGGTT**
CUCCCACAAGGGAAGGGGA	**Mapk4 #3**	**CUCCCACAAGGGAAGGGGATT**	**UCCCCUUCCCUUGUGGGAGTT**
UUCUAAAAUAGUGGCUAUU	**Psd3 #1**	**UUCUAAAAUAGUGGCUAUUTT**	**AAUAGCCACUAUUUUAGAATT**
UCUUCUAAAAUAGUGGCUA	**Psd3 #2**	**UCUUCUAAAAUAGUGGCUATT**	**UAGCCACUAUUUUAGAAGATT**
GAUCUUCUAAAAUAGUGGC	**Psd3 #3**	**GAUCUUCUAAAAUAGUGGCTT**	**GCCACUAUUUUAGAAGAUCTT**
AGAUGUAGGCUCCGUGCUC	**Gigyf2 #1**	**AGAUGUAGGCUCCGUGCUCTT**	**GAGCACGGAGCCUACAUCUTT**
GAUGUAGGCUCCGUGCUCU	**Gigyf2 #2**	**GAUGUAGGCUCCGUGCUCUTT**	**AGAGCACGGAGCCUACAUCTT**
AUGUAGGCUCCGUGCUCUG	**Gigyf2 #3**	**AUGUAGGCUCCGUGCUCUGTT**	**CAGAGCACGGAGCCUACAUTT**
CUCAGCAGGUUUGGGCCCC	**pwwp2a #1**	**CUCAGCAGGUUUGGGCCCCTT**	**GGGGCCCAAACCUGCUGAGTT**
CGCUCAGCAGGUUUGGGCC	**pwwp2a #2**	**CGCUCAGCAGGUUUGGGCCTT**	**GGCCCAAACCUGCUGAGCGTT**
CCCGCUCAGCAGGUUUGGG	**pwwp2a #3**	**CCCGCUCAGCAGGUUUGGGTT**	**CCCAAACCUGCUGAGCGGGTT**
GCAAAGGCGUAUUCUGCAC	**Upf2 #1**	**GCAAAGGCGUAUUCUGCACTT**	**GUGCAGAAUACGCCUUUGCTT**

AGGCAAAGGCGUA UUCUGC	**Upf2 #2**	**AGGCAAAGGCGUA UUCUGCTT**	**GCAGAAUACGCCU UUGCCUTT**
GGCAAAGGCGUAU UCUGCA	**Upf2 #3**	**GGCAAAGGCGUAU UCUGCATT**	**UGCAGAAUACGCC UUUGCCTT**
UGCCGUAUCCAGG GGUUUC	**Cdr1as #1**	**UGCCGUAUCCAGG GGUUUCTT**	**GAAACCCCUGGAU ACGGCATT**
CCGUAUCCAGGGG UUUCCA	**Cdr1as #2**	**CCGUAUCCAGGGG UUUCCATT**	**UGGAAACCCCUGG AUACGGTT**
GUAUCCAGGGGUU UCCAGU	**Cdr1as #3**	**GUAUCCAGGGGUU UCCAGUTT**	**ACUGGAAACCCCU GGAUACTT**
CUAUAGGUAUGGC CUCACA	**Hipk3 #1**	**CUAUAGGUAUGGC CUCACATT**	**UGUGAGGCCAUAC CUAUAGTT**
GGUACUAUAGGUA UGGCCU	**Hipk3 #2**	**GGUACUAUAGGUA UGGCCUTT**	**AGGCCAUACCUAU AGUACCTT**
UACUAUAGGUAUG GCCUCA	**Hipk3 #3**	**UACUAUAGGUAUG GCCUCATT**	**UGAGGCCAUACCU AUAGUATT**

Silencer Select Negative Control #1 (Catalog number 4390843, Thermo Fisher), Silencer Select Negative Control#2 (Catalog number 4390847, Thermo Fisher) and a mixture of 50/50 “siControl Pool”, were utilised in the siRNA knockdown screen. These are referred to as siControl1, siControl2 and siControl Pool in the text.

shRNAs:

“pSUPER” plasmid utilised as a control, all nucleotides excised between BglII and HindII RE sites, preventing shRNA production.

For additional shRNAs below are listed sense sequences:

ScramSh: AAACCTTGTGGTCCTTAGG

linRERE

sh1 RERE: GGAACGAGAGCGAGAGAAA (RERE exon19)

sh2 RERE: CCAAGAAAGTGAAGGAAGA (RERE exon16-17)

sh3 RERE: ACAGAAAGCCCGAGAGGAA (RERE exon 19)

circRERE2

Sh1: ATGAAGTGAGTAAGAGGGA

Sh2: GAAGTGAGTAAGAGGGACC

Sh3: GGATGAAGTGAGTAAGAGG

Note: circRERE2Sh2 noted above was utilised for all experiments after validation of efficacy and specificity.

circRERE1 Sh

AACACAAGUAAGAGGGACC

circRERE3 Sh

AGGAAACCAGUAAGAGGGA

circRNA FISH Probes

Target sequences for probe design (rat)
circHomer1: 5’-UUUCACAUAGGGAACAACCU-3’
circRmst_2: 5’-GGCUUCAUGAGAUACUCAUG-3’

circStau2_1: 5’- ACAACCAGAGUUUUUUGGAG -3’

circRERE_2: 5’- GGAUGAAGUGAGUAAGAGGG -3’

miR-128-3p Perfect binding site reporter insert

gctagcAAAGAGACCGGTTCACTGTGActAAAGAGACCGGTTCACTGTGgtcgac

Control Reporter possessed no insert between NheI/SalI RE sites.
