# Supplementary material for: A functional screen uncovers circular RNAs regulating excitatory synaptogenesis in hippocampal neurons
Source: Nat Commun. 2025 Mar 28;16:3040. doi: 10.1038/s41467-025-58070-4 (PMC11953392; doi:10.1038/s41467-025-58070-4)
Supplement: Supplementary file 3 — Reporting Summary [file 41467_2025_58070_MOESM3_ESM.pdf]

nature portfolio

Corresponding author(s): Dr. Gerhard Schratt
Last updated by author(s): 29/01/25

Reporting Summary

Nature Portfolio wishes to improve the reproducibility of the work that we publish. This form provides structure for consistency and transparency in reporting. For further information on Nature Portfolio policies, see our Editorial Policies and the Editorial Policy Checklist.

Statistics

For all statistical analyses, confirm that the following items are present in the figure legend, table legend, main text, or Methods section.

n/a	Confirmed	
☐	☒	The exact sample size (n) for each experimental group/condition, given as a discrete number and unit of measurement
☐	☒	A statement on whether measurements were taken from distinct samples or whether the same sample was measured repeatedly
☐	☒	The statistical test(s) used AND whether they are one- or two-sided<br>*Only common tests should be described solely by name; describe more complex techniques in the Methods section.*
☒	☐	A description of all covariates tested
☐	☒	A description of any assumptions or corrections, such as tests of normality and adjustment for multiple comparisons
☐	☒	A full description of the statistical parameters including central tendency (e.g. means) or other basic estimates (e.g. regression coefficient) AND variation (e.g. standard deviation) or associated estimates of uncertainty (e.g. confidence intervals)
☐	☒	For null hypothesis testing, the test statistic (e.g. F, t, r) with confidence intervals, effect sizes, degrees of freedom and P value noted<br>*Give P values as exact values whenever suitable.*
☒	☐	For Bayesian analysis, information on the choice of priors and Markov chain Monte Carlo settings
☒	☐	For hierarchical and complex designs, identification of the appropriate level for tests and full reporting of outcomes
☒	☐	Estimates of effect sizes (e.g. Cohen's d, Pearson's r), indicating how they were calculated

Our web collection on statistics for biologists contains articles on many of the points above.

Software and code

Policy information about availability of computer code

Data collection	Data was collected in this study primarily as microscopy images gathered through ZEN software on the form of CZI images and processed and analyzed using previously published and adapted FIJI based image analysis pipelines described below. Full details of tools used are descibed in the Materials and Methods segment of the main text. List: EnrichMir version 0.99.28 https://ethz-ins.org/enrichMiR. Graphpad Prism 9. ScanMiR webtool version 1.5.2 https://ethz-ins.org/scanMiR/. R/ggplot2. ImageJ/FIJI 2.16.0. fgsea package v1.160. TopGo algorithm (v.2.52.0). clusterProfiler 4.10.1 .
Data analysis	All scripts and R markdowns for analytical pipelines for cell morphological analysis available upon request in detail. FIJI pipeline templates utilised in this study for the analysis of synapse co-cluster and dendritic spine density are available at https://github.com/dcolam/Cluster-Analysis-Plugin. Zenodo. doi: 10.5281/zenodo.8167635

For manuscripts utilizing custom algorithms or software that are central to the research but not yet described in published literature, software must be made available to editors and reviewers. We strongly encourage code deposition in a community repository (e.g. GitHub). See the Nature Portfolio guidelines for submitting code & software for further information.

Data

Policy information about availability of data

All manuscripts must include a data availability statement. This statement should provide the following information, where applicable:

- Accession codes, unique identifiers, or web links for publicly available datasets
- A description of any restrictions on data availability
- For clinical datasets or third party data, please ensure that the statement adheres to our policy

RNA sequencing data has been deposited to Gene Expression Omnibus (GEO, Poly-A sequencing Fig. 5A, Small-RNA sequencing Fig 6A, accession number GSE261610). The images/data that support the findings of this study are available from the corresponding author upon request.

Research involving human participants, their data, or biological material

Policy information about studies with human participants or human data. See also policy information about sex, gender (identity/presentation), and sexual orientation and race, ethnicity and racism.

Reporting on sex and gender	*Use the terms sex (biological attribute) and gender (shaped by social and cultural circumstances) carefully in order to avoid confusing both terms. Indicate if findings apply to only one sex or gender; describe whether sex and gender were considered in study design; whether sex and/or gender was determined based on self-reporting or assigned and methods used. Provide in the source data disaggregated sex and gender data, where this information has been collected, and if consent has been obtained for sharing of individual-level data; provide overall numbers in this Reporting Summary. Please state if this information has not been collected. Report sex- and gender-based analyses where performed, justify reasons for lack of sex- and gender-based analysis.*
Reporting on race, ethnicity, or other socially relevant groupings	*Please specify the socially constructed or socially relevant categorization variable(s) used in your manuscript and explain why they were used. Please note that such variables should not be used as proxies for other socially constructed/relevant variables (for example, race or ethnicity should not be used as a proxy for socioeconomic status). Provide clear definitions of the relevant terms used, how they were provided (by the participants/respondents, the researchers, or third parties), and the method(s) used to classify people into the different categories (e.g. self-report, census or administrative data, social media data, etc.) Please provide details about how you controlled for confounding variables in your analyses.*
Population characteristics	*Describe the covariate-relevant population characteristics of the human research participants (e.g. age, genotypic information, past and current diagnosis and treatment categories). If you filled out the behavioural & social sciences study design questions and have nothing to add here, write "See above."*
Recruitment	*Describe how participants were recruited. Outline any potential self-selection bias or other biases that may be present and how these are likely to impact results.*
Ethics oversight	*Identify the organization(s) that approved the study protocol.*

Note that full information on the approval of the study protocol must also be provided in the manuscript.

Field-specific reporting

Please select the one below that is the best fit for your research. If you are not sure, read the appropriate sections before making your selection.

☒ Life sciences ☐ Behavioural & social sciences ☐ Ecological, evolutionary & environmental sciences

For a reference copy of the document with all sections, see nature.com/documents/nr-reporting-summary-flat.pdf

Life sciences study design

All studies must disclose on these points even when the disclosure is negative.

Sample size	Our work in primary neuronal cultures typically utilised 4 independent biological replicates with cells sourced from different animal sources. Within these biological replicates, multiple technical replicates (individual neurons) were measured and weighted appropriated in described linear models. RNA-Seq analysis also utilised 4 distinct biological replicates from different primary neuron preparations.
Data exclusions	A biological replicate was excluded if the health of the neuronal culture did not meet quality standards and possess sufficient transfected neurons before microscopy analysis.
Replication	Replication of key results involved the utilisation of multiple biological and technical replicates as described. Key findings such as miRNA levels were validated with multiple approaches. e.g. small RNA-seq, luciferase assay, miRNA FISH and enrichMir analysis.
Randomization	Microscopy work was blinded and took place in a random order to prevent bias.
Blinding	Slides were blinded before microscopy analysis and unblinded after image analysis.

Reporting for specific materials, systems and methods

We require information from authors about some types of materials, experimental systems and methods used in many studies. Here, indicate whether each material, system or method listed is relevant to your study. If you are not sure if a list item applies to your research, read the appropriate section before selecting a response.

Materials & experimental systems

n/a	Involved in the study
☐	☒ Antibodies
☒	☐ Eukaryotic cell lines
☒	☐ Palaeontology and archaeology
☐	☒ Animals and other organisms
☒	☐ Clinical data
☒	☐ Dual use research of concern
☒	☐ Plants

Methods

n/a	Involved in the study
☒	☐ ChIP-seq
☒	☐ Flow cytometry
☒	☐ MRI-based neuroimaging

Antibodies

Antibodies used	Rabbit polyclonal anti-GluA1, 1:1000 (PC246 Calbiochem EMD Biosciences), rabbit anti-Synapsin1, 1:1000 (AB1543, Merck Millipore), and mouse anti-PSD-95, 1:200 (810401; BioLegend).
Validation	GluA1 validated in rat: Xu, Z.X., Tan, J.W., Xu, H., Hill, C.J., Ostrovskaya, O., Martemyanov, K.A. and Xu, B., 2019. Caspase-2 promotes AMPA receptor internalization and cognitive flexibility via mTORC2-AKT-GSK3β signaling. Nature communications, 10(1), p.3622.<br><br>Synapsin1 validated in rat: Ribes, J.M., Patel, M.P., Halim, H.A., Berretta, A., Tooze, S.A. and Klöhn, P.C., 2023. Prion protein conversion at two distinct cellular sites precedes fibrillisation. Nature Communications, 14(1), p.8354.<br><br>PSD95 validated in rat (Glua1 and Syn1 also validated): Inouye, M.O., Colameo, D., Ammann, I., Winterer, J. and Schratt, G., 2022. miR-329–and miR-495–mediated Prr7 down-regulation is required for homeostatic synaptic depression in rat hippocampal neurons. Life Science Alliance, 5(12).

Animals and other research organisms

Policy information about studies involving animals; ARRIVE guidelines recommended for reporting animal research, and Sex and Gender in Research

Laboratory animals	Sprague-Dawley rats (Janvier Laboratories)
Wild animals	N/A
Reporting on sex	This work primarily utilised mixed primary neuronal cultures from both male and female embryos and thus the main findings do not distinguish by sex. RNA collected for qPCR validations in adult rats are from females.
Field-collected samples	N/A
Ethics oversight	All animal experiments were<br><br>performed in accordance with the animal protection law of Switzerland and were approved by the<br><br>local cantonal authorities (license nr.ZH027/2021 and nr.ZH112/2024)

Note that full information on the approval of the study protocol must also be provided in the manuscript.

Plants

Seed stocks	*Report on the source of all seed stocks or other plant material used. If applicable, state the seed stock centre and catalogue number. If plant specimens were collected from the field, describe the collection location, date and sampling procedures.*
Novel plant genotypes	*Describe the methods by which all novel plant genotypes were produced. This includes those generated by transgenic approaches, gene editing, chemical/radiation-based mutagenesis and hybridization. For transgenic lines, describe the transformation method, the number of independent lines analyzed and the generation upon which experiments were performed. For gene-edited lines, describe the editor used, the endogenous sequence targeted for editing, the targeting guide RNA sequence (if applicable) and how the editor was applied.*
Authentication	*Describe any authentication procedures for each seed stock used or novel genotype generated. Describe any experiments used to assess the effect of a mutation and, where applicable, how potential secondary effects (e.g. second site T-DNA insertions, mosiacism, off-target gene editing) were examined.*
